# Ruler Arrays Reveal Haploid Genomic Structural Variation

**DOI:** 10.1371/journal.pone.0043210

**Published:** 2012-08-27

**Authors:** P. Alexander Rolfe, Douglas A. Bernstein, Paula Grisafi, Gerald R. Fink, David K. Gifford

**Affiliations:** 1 Computer Science and Artificial Intelligence Laboratory, Massachusetts Institute of Technology, Cambridge, Massachusetts, United States of America; 2 The Whitehead Institute for Biomedical Research, Cambridge, Massachusetts, United States of America; Centro Cardiologico Monzino IRCCS, Italy

## Abstract

Despite the known relevance of genomic structural variants to pathogen behavior, cancer, development, and evolution, certain repeat based structural variants may evade detection by existing high-throughput techniques. Here, we present ruler arrays, a technique to detect genomic structural variants including insertions and deletions (indels), duplications, and translocations. A ruler array exploits DNA polymerase’s processivity to detect physical distances between defined genomic sequences regardless of the intervening sequence. The method combines a sample preparation protocol, tiling genomic microarrays, and a new computational analysis. The analysis of ruler array data from two genomic samples enables the identification of structural variation between the samples. In an empirical test between two closely related haploid strains of yeast ruler arrays detected 78% of the structural variants larger than 100 bp.

## Introduction

Although single nucleotide polymorphisms (SNPs) are thought to play a significant role in phenotypic distinction, recent whole genome comparisons suggest that structural variants (insertions, deletions, duplications, translocations, and inversions) also play important roles in the distinction between species, strains, and even individuals [1]–[6]. In addition, these structural variants correlate with many diseases and such genome instabilities are an underlying characteristic of cancer [7].

Despite the importance of structural variants, current technologies cannot reliably detect all variants. PCR can only monitor a limited number of variants per reaction, limiting the number loci it can feasibly monitor.

Array-based comparative genome hybridization (aCGH) requires two genomic samples labeled with different fluorophores and can detect copy number changes but not necessarily the site of the change [8]–[15]. The two samples are hybridized to a single microarray and scanned. Comparing the intensities in the two channels at each probe or set of genomicaly proximal probes determines the presence of duplications (higher intensity than expected compared to other probes in the same sample or compared to the other sample) and deletions (lower than expected intensities). While the location of a deletion in the genome is apparent if one knows the genomic location of the relevant probes and if the deletion removes enough probes from the sample sequence, aCGH does not provide the genomic location of duplications. Furthermore, aCGH cannot necessarily detect rearrangements, though very high density arrays may be able to detect candidate rearrangements when low intensity probes, those spanning the relocation boundary, surround probes of the expected intensity.

Paired-end high-throughput sequencing permits many forms of structural variants to be discovered by detecting deviations from the expected distance between ends [16], [17]. In this technique, often called Paired End Mapping (PEM), a sequencing library is generated by randomly shearing DNA fragments. The library can be described by the mean and variance of the fragment lengths. The ends of the fragments are sequenced and mapped to a reference genome and the analysis looks for sites spanned by pairs of reads whose average mapped distance differs from that of the library as a whole. A deletion in the experimental genome presents as reads that map far apart in the reference genome; an insertion presents as reads that map nearby. A analysis compares the observed average distance between ends spanning some site to the predicted and tests for a significant difference. Thus, control of the variance of the fragment lengths is key to PEM. High variance reduces the power of the technique to observe indels. Either by control of the shearing or by size selection of the product, PEM seeks to limit the variance of the fragment lengths. Depending on the fragment lengths, PEM may also require difficult circularization protocols that limit read lengths and thus mappability (eg circularization followed by digestion with MmeI) [18].

Sequencing has the potential to detect all sequence changes, but its practical limitations depend on the technology used (which determines the read length, the availability of paired reads, and the mean and standard deviation of the distance between pairs of reads) and the coverage depth. In general, all sequencing approaches can detect SNPs since single nucleotide changes are small compared even to the short reads produced by current high throughput techniques [19], [20].

Sequencing and assembly based approaches cannot resolve differences in periodic structures that are not spanned by sequenced fragments [1], [21]. For example, consider a repetitive element of length 1kb. No read in a 300 bp shotgun library will span the element, thus producing a contig boundary at every instance of the element. While long fragments, contigs, and scaffolds are available with various circularization protocols or techniques such and BACs, these techniques are difficult and not routinely applied to most new genome projects. In addition, even high quality genome assemblies may produce ambiguous results near repetitive elements [22], [23].

To monitor the plasticity of both repetitive and nonrepetitive elements of genomes we have developed a microarray technology called a ruler array that measures the distance between pairs of defined sequences in a genome. Every microarray probe sequence defines one end of a ruler with the other end defined by a sequence feature such as a restriction site. The ruler measures the distance between the two ends and thus can detect structural changes in the intervening sequence. Thus ruler arrays can detect changes in the size of repetitive sequences that are proximal to a unique probe sequence and a suitable restriction site. As such, the ruler array offers a complement to sequencing based approaches for structural variant discovery.

## Results

**Figure 1:**
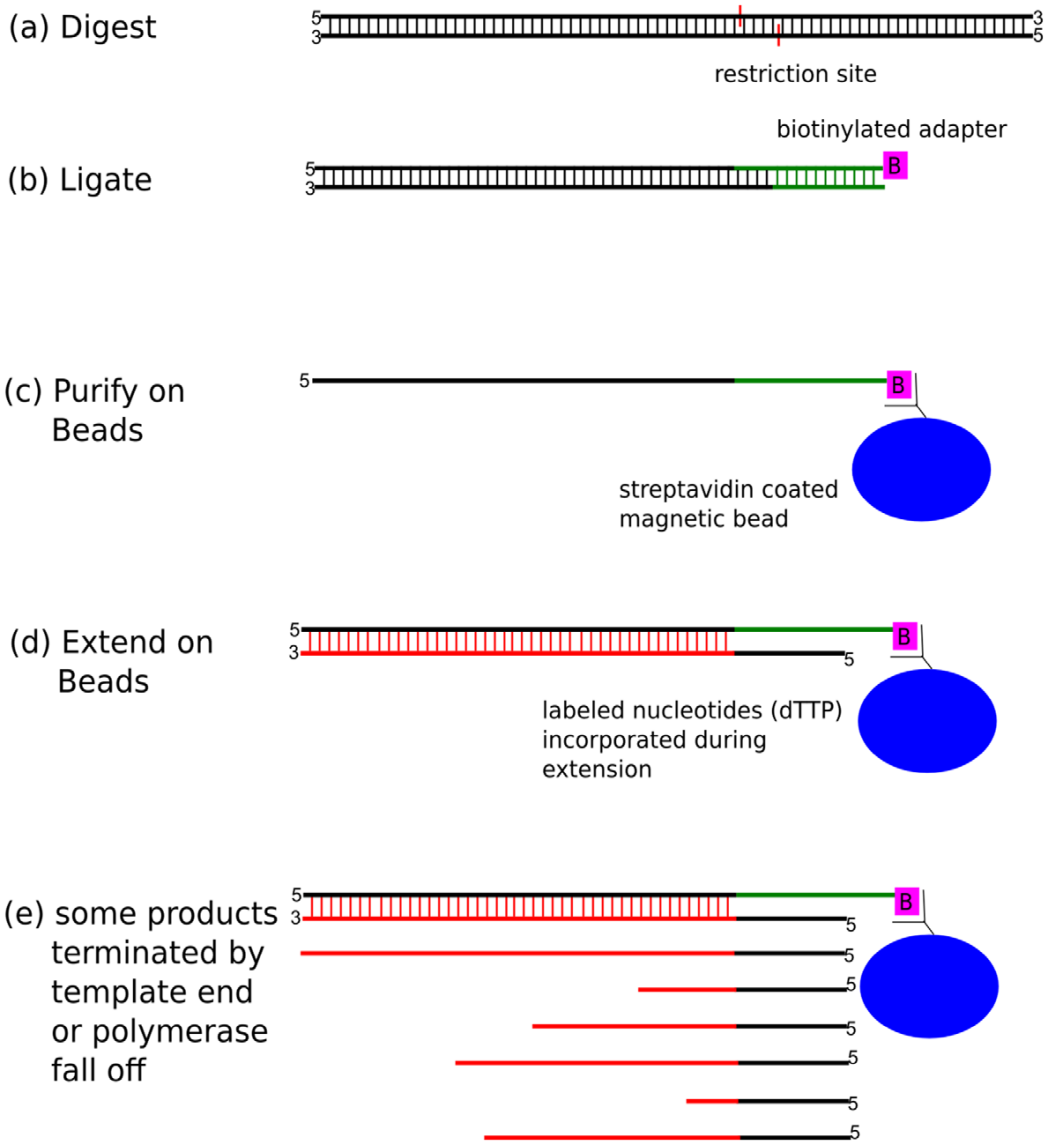
The ruler array method uses a digest-ligate-extend protocol to generate a labeled DNA sample to detect distances between genomic points. (a) One or more restriction enzymes digest the input DNA sample, leaving a set of sticky ends. (b) An adapter DNA molecule is ligated to the ends, providing a biotin moiety for purification of the ligated material and an initiation site for the polymerase extensions. (c) The ligated material is purified using streptavidin coated beads. (d) Primers, DNA polymerase, and labeled nucleotides are added and primer extensions occur. (e) An extension terminates either upon reaching the end of the template molecule or randomly due to the polymerase’s processivity. Since the output material includes many partial extension products, sequences close to the restriction site occur more frequently than do sequences far from the restriction site. When the labeled sample is hybridized to the microarray, probes close to the restriction site yield correspondingly higher intensities than the distal probes. When this material is labeled (the polymerase may incorporate labeled bases or modified bases or the product may be labeled with a system like ULS) and hybridized to a microarray, probes near the restriction site in the genome will observe a high intensity while probes farther away observe lower intensities.

**Figure 2:**
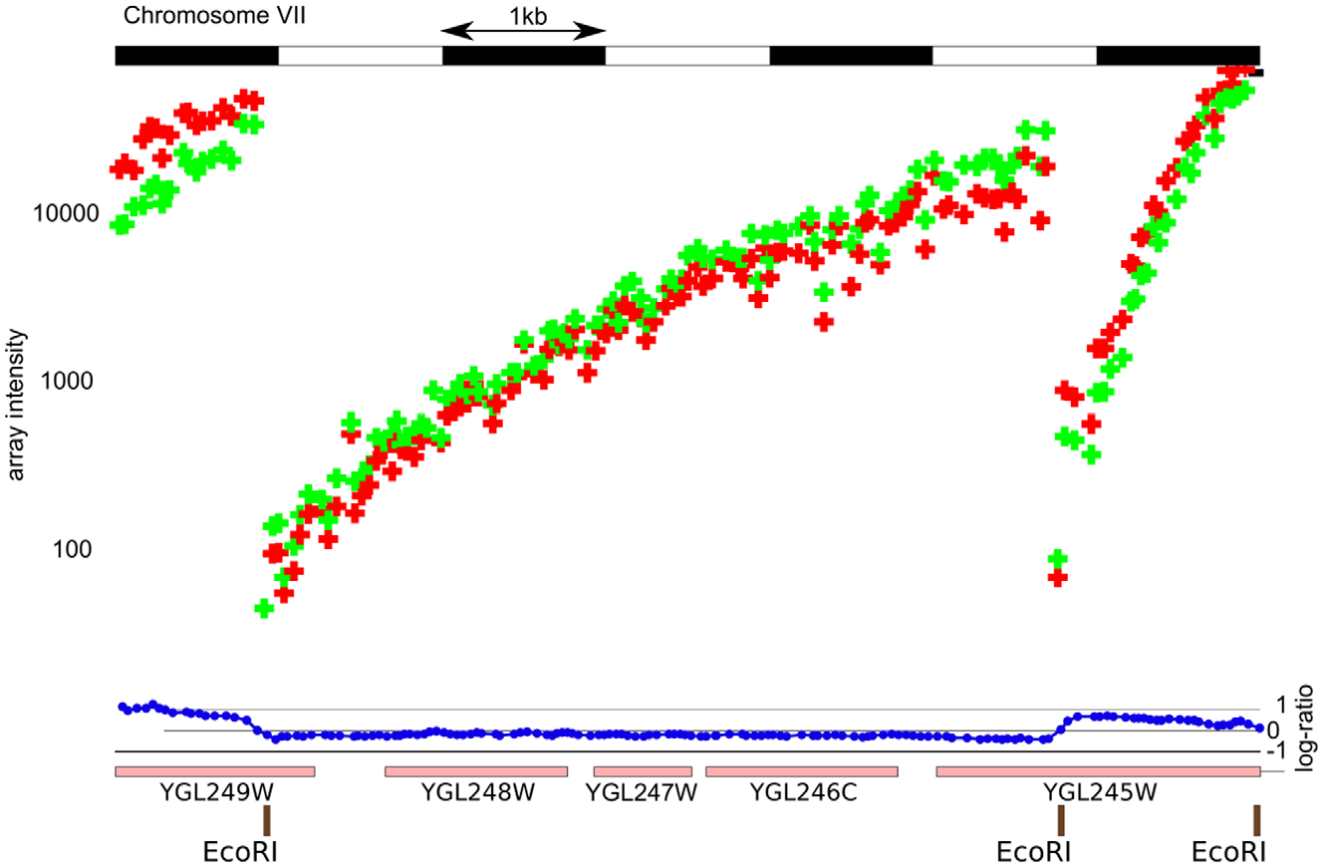
Log-intensities from a ruler array experiment over part of chromosome VII demonstrate the log-linear decrease in observed array intensity as distance increases from the restriction site. The red marks indicate probe observations from the S288c channel and the green marks indicate observations from the 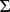
1278b channel. Note the similarity between the channels (blue marks towards bottom show the ratio between channels) and the relatively log-linear fall off between the restriction sites (brown tick marks at bottom). Note that the intensities are highest over the restriction sites (tick marks at bottom) and fall off roughly linearly.

The ruler array protocol generates a population of labeled DNA fragments where the probability that the population contains a specific sequence is inversely related to the sequence’s physical distance to a selected restriction site (Figure 1). When the labeled material is hybridized to a microarray, probe sequences proximal to restriction sites yield correspondingly higher intensities than distal probe sequences (Figure 2). The observed intensity falloff is roughly log-linear and consistent with a model in which the extension terminates with equal probability at each base (Figure S1). The ruler array protocol generates this population of fragments by first digesting a genomic sample with a restriction enzyme and ligating an adapter to the resulting ends. Polymerase extensions are then initiated from a primer that is complementary to the adapter, producing many copies of sequence proximal to the adapter but fewer copies of distal sequence as the limits of processivity for the polymerase are approached and it stochastically terminates.

**Figure 3:**
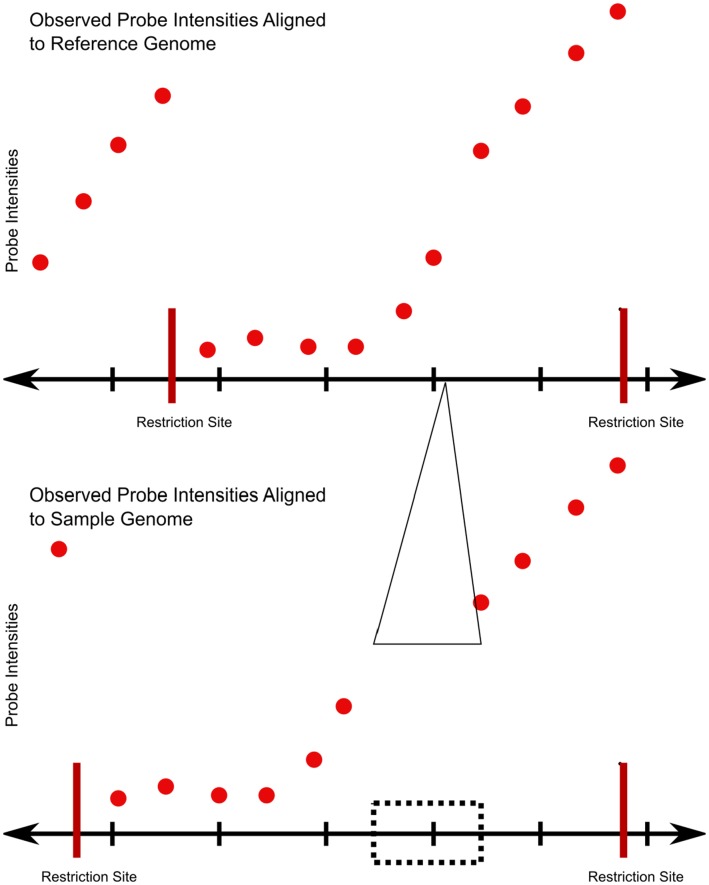
Schematic ruler array probe intensities at an insertion (top) show a drop over the insertion site. When probe intensities from a strain with an insertion are mapped to the reference genome, the intensities drop at the insertion site relative to the log-linear falloff over sequence that contains no indels. The bottom track maps the observed probe intensities to the strain from which the sample was generated, showing the expected linear falloff. In our protocol, samples from both strains are hybridized such that the analysis method can use the reference strain intensities to account for noise.

**Figure 4:**
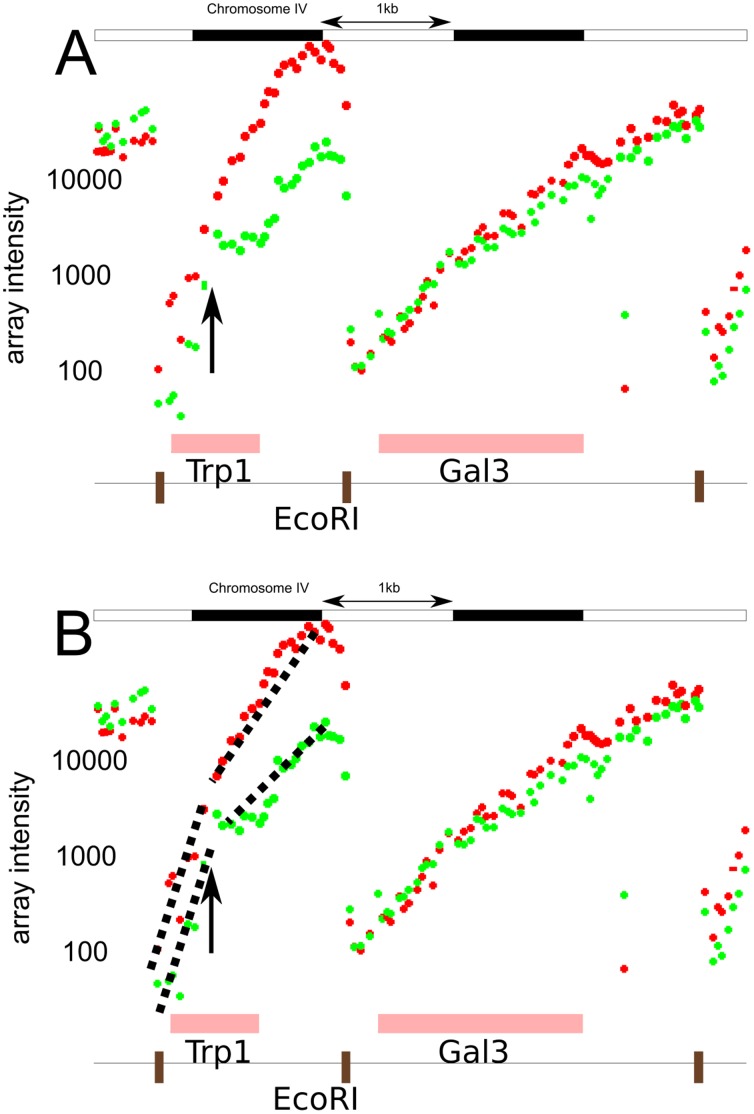
The ruler array analysis recognizes structural variants by fitting line segments to the microarray data and detecting differences in those segments between channels. (a) The observed Ruler Array intensities (red is S288c and green is 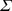
1278b) at *TRP1* reveal the presence of the synthetic 1182 bp insertion (bacterial HISG) in this 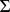
1278b *trp-* strain. The adjacent segment, which contains GAL3 and no differences between 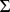
1278b and S288c, shows no differences between strains and the two channels’ intensities track very closely to each other. (b) The dashed black lines show the fitted segments at *TRP1*, emphasizing the insertion’s position by the difference in the segments fitted to the two channels. The sequences of the tiling array probes are from the S288c genome.

Comparing ruler array hybridization data from two genomic samples reveals differences between the corresponding genomes (Figures 3 and S2). When a sequence is farther from the restriction site in one genome than the other, the observed probe intensities beyond that sequence will be lower in the corresponding channel. Thus, a discontinuity in a line fitted to the intensities in one channel and the absence of a discontinuity in the intensities of the other indicates a sudden jump in the distance of the probes from the restriction site (Figure 4). The intensity drop does not generally depend on the content of the insertion or deletion, only the change in distance between genomic points.

We compared the genomes of the *S. cerevisiae* haploid yeast strains S288c and 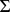
1278b [24] using ruler arrays with strain specific genome assemblies serving as a control. Ruler array performance was calculated by comparing ruler array variant predictions to two sets of assembly-derived predictions. During curation of the long read 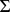
1278b assembly, we selected 106 apparent indels of more than 100 bp relative to S288c for PCR confirmation. These indels were identified by several alignment programs (FSA [25], Blast [26], Blat [27], custom code) and by manual inspection of the alignment results. Thirty-six of the 106 resulted in PCR gel bands whose length differed by roughly 100 bp or more, giving a false positive rate of 66% for the early 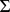
1278b assembly (Table S1 lists the confirmed changes). We detected a total of 114 additional indels between the genomes beyond the 106 selected for confirmation based on the final 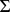
1278b assembly.

Two ruler array replicates identified roughly 75% of the PCR confirmed changes (28 and 25 of 36) and many (28 and 20 out of 114) of the set of 100 bp changes. Due to noise and protocol variations between the replicates (such as the polymerase used), the two replicates discover similar but not identical sets of indels and their intersection represents a set of high quality calls. The two replicates also generated a number of false positive calls, predictions that do not correspond to a change of more than 4 bp. There were 553 false positives for the first replicate and 414 for the second.

We used a single replicate of an aCGH experiment between FY4 and 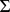
1278b to compare aCGH’s performance against that of the Ruler Array. The experimental protocol used the non-enzymatic ULS labeling system to avoid amplification or dye incorporation biases.

Our HMM analysis of the aCGH experiment produced 183 calls. Twelve appear incorrect given the two genome assemblies and 33 are confirmed by the assemblies. The remainder occur in repetitive regions (e.g. TY, sigma, tau, and delta elements) such that both the CGH data and the assembly are likely to be incorrect.

**Figure 5:**
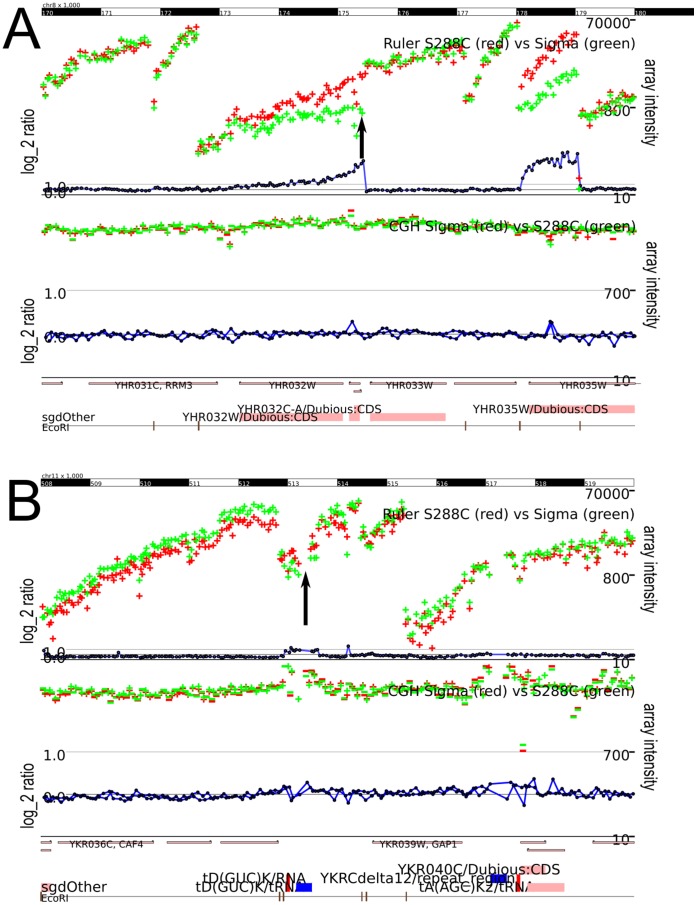
The ruler array can detect structural variants that array-CGH misses. (A) The ruler array (data in top track) successfully detects the insertion of roughly 100 bp on chromosome eight while the unique probes in the aCGH data show no difference. The red and green points show the channel intensities and the blue line shows the log-ratio. (B) The Ruler Array (data in top track) successfully detects the insertion of a TY element on chromosome eleven while the unique probes in the aCGH data show no difference. While the CGH data does show a difference in ratio over repetitive elements such as the TY family, it cannot localize the changes to particular insertion sites such as this one. In the aCGH plot, the FY4 intensities are green and the 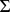
1278b intensities are red; the ratio is shown in blue. Both methods clearly show a deletion in 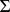
1278b at the left edge of the plot.

The aCGH experiment found 21 of the 35 “must-find” indels and missed the remaining 14. Thirteen of the 35 were originally added to our list of known indels because of the aCGH experiment, so their detection is not surprising. Figure 5 shows two examples of insertions that the aCGH experiment misses because there is no change in the unique probes surrounding the changes.

To more accurately compare the aCGH experiment to the Ruler Array experiment, we re-ran the analysis using only array probes with a unique genomic location; this excludes probes that map to TY or other repetitive elements. By only including unique probes, we now know the location of any change that the aCGH experiment detects. On this input, the same HMM analysis produced only 18 calls and found 6 of the 35 “must find” events.

**Figure 6:**
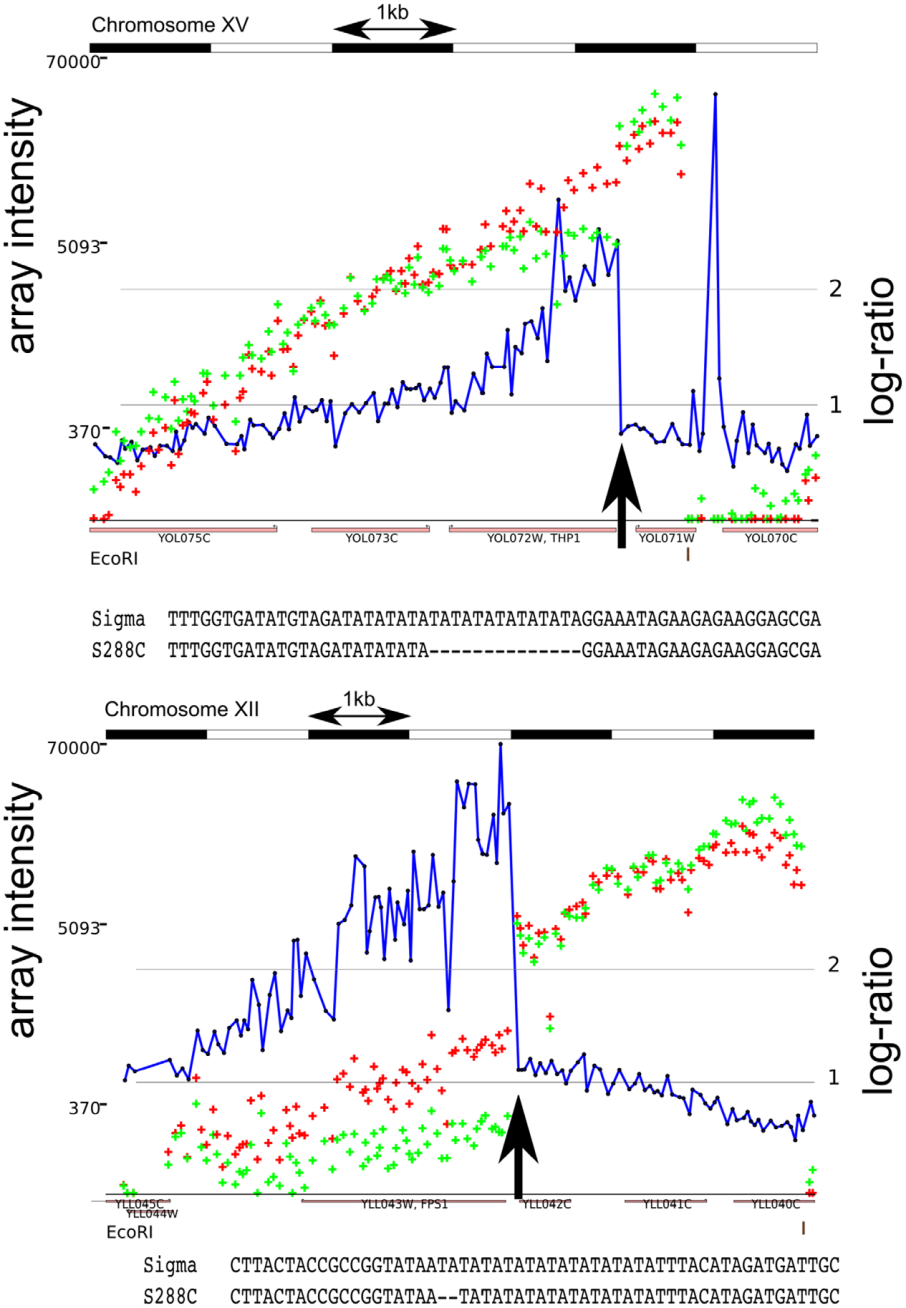
Two examples of Ruler Array data (S288c in red, 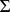
1278b in green) and genomic sequence demonstrating the impact of AT repeat length changes on polymerase processivity. The 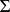
1278b intensities fall suddenly over the repeat. Both examples were sequenced in both directions to confirm the repeat length difference, 14 bp in the upper example and 2 bp in the lower.

Our ruler array experiments comparing S288c to 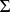
1278b revealed non-uniform polymerase processivity at particular sequence elements. Poly A, AT, or AAT repeats, often found at transcription stop and start sites [28], sometimes caused rapid termination of the polymerase extension and a corresponding drop in observed intensity. In many cases, a small change in the length of such a repeat sequence leads to a discontinuity in the ruler array signal such as one might expect from a large insertion. Thus, we detect certain insertions and deletions as small as 2 bp when they occur in these repeats. Figure 6 shows two such examples. These repeats may also cause reduced signal in downstream sequence.

## Discussion

We have shown that the ruler array technology can detect structural variants between two closely related strains of haploid yeast, detecting changes of more than 100 bp with high frequency. We found that ruler arrays can fail to detect structural variants between haploid genomes for three reasons. First, a variant may be too close or too far from the restriction site being used such that the variant’s discontinuity is not detectable. Second, an insertion may carry a restriction site that counters an expected intensity drop. Third, the ruler array may miss changes in poorly tiled regions of the genome such as the telomeres or long clusters of repetitive elements.

Diploid genomes present challenges for the ruler array as the signals from the two chromosomes will be averaged, thus making detection more difficult. Furthermore, the two chromosomes might contain different restriction sites due to SNPs, generating additional signal that may be difficult to interpret.

The ruler array obviously depends on the characteristics of the polymerase. As mentioned, certain sequence elements cause frequent termination. On normal sequence, the polymerase’s processivity determines the slope of the decrease in signal over distance. A polymerase with poor processivity generates a higher slope and thus a greater change in observed intensity for a given indel than a more processive polymerase. However, our experience indicates that the benefits of the higher slope are outweighed by the fact that fewer microarray probes observe the change in intensity (the higher slope means the signal reaches background over less distance). Other characteristics of the polymerase, such as priming efficiency, may influence the overall efficiency of the reaction and the maximum signal level. These characteristics may change the sensitivity of the method but are unlikely to generate spurious calls as they would effect both channels equally.

We believe the ruler array offers a novel and potentially useful technology for surveying related genomes for structural changes in both repetitive and non-repetitive DNA elements. While the current protocol suffers from a high false positive rate, it offers an effective complement to sequencing-based approaches. Further refinement could lead to the development of a ruler array based sequencing assay as a proofreading technique to check newly assembled genomes for large structural variants indicative of misassembly.

## Materials and Methods

### Adapter Design

The adapter (and therefore primer) sequences were chosen to match the overhang left by EcoRI, have minimal genomic matches in yeast, and have a melting temperature suitable for the polymerase extensions. The sequence used was

5′ P-AATTGGAGGAGGGAAGGGGG-B 3′

3′ CCTCCTCCCTTCCCCC 5′.

where P indicates a 5′ phosphate (necessary for the ligation) and B indicates a 3′ biotin to allow purification of the ligated material from the remainder of the reaction mix.

Note that the shorter oligo serves both as part of the adapter (in the ligation reaction) and as the primer in the polymerase extensions.

### Sample Preparation

The ruler array protocol begins with 20 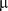
g of pure *S. cerevisiae* genomic DNA prepared using standard laboratory protocols. The DNA sample was digested using a suitable restriction enzyme (eg, EcoRI). Digested DNA was then treated with Calf Intestinal Phosphatase (CIP) to remove the 5′ phosphate from the digested products in turn preventing genome reassembly during adapter addition. Phenol Chloroform extraction and ethanol precipitation removed restriction enzymes and CIP. Purified digested genomic DNA was ligated to 1.6 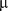
M of biotin labeled primer pair for 16 hours at 16°C. Ligation products were bound to Streptavidin beads using the Dynabeads kilobase Binder kit. Polymerase extension reactions were initiated with Vent Exo- or ExTaq polymerase from the ligated primer. Aminoallyl dUTP are included at a concentration of .3 mM in the extension reactions and the DNA extension products were labeled with Alexa Fluor reactive dyes using established protocols. Labeled DNA is purified using a QIAGEN spin column. Dye incorporation was assessed by nanodrop and DNA containing between 30 and 100 pmol of dye was applied to a Agilent yeast whole genome array (238 k non-control 60 bp probes, Agilent microarray design 147411). After incubating at 65°C for 40 hours arrays were scanned using Agilent DNA Microarray Scanner and images were feature extracted using Agilent feature extraction software.

### Data Processing

Probes were mapped to the October 2006 S288c reference genome from the Stanford Genome Database using Blat [27]. We retained probes that had only a single hit of more than 50 bp; probes mapping to multiple locations were ignored. We normalized the two channels to each other by multiplying the Cy5 intensities by median(Cy3)/median(Cy5) to account for differences in the amount of dye hybridized in each channel. We further normalized by computing the regression line of the log-transformed Cy5 values on the log-transformed Cy3 values and then rotating the log values such that this line is the diagonal.

Our analysis method performs simultaneous segmentation and linefitting on the log-transformed intensities. A basic, single-channel segmentation and linefitting procedure minimizes
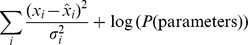
where 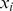 is the log-transformed intensity observation, 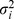 is the estimated variance for the observation, and the probability of the parameters penalizes the use of more segments. The estimated variance depends on the local co-linearity of the log-transformed intensities and a term based on the intensity.

We extended this segmentation and linefitting method to handle both channels simultaneously and incorporate prior probabilities of using the same segment boundaries in both channels and to using the same segment slopes in both channels (we found that using the same intercept yielded worse performance than constraining only the slope). The analysis of the resulting fitted segments calls an indel at a segment boundary that exists in one channel but not the other or at segment boundaries at which the intensity change differs substantially between channels (Figure S3). The supporting methods (Text S1) present a more thorough description of the normalization and analysis technique.

The ruler array analysis software and sample data are available at http://cgs.csail.mit.edu/rulers/.

### Data and Code

The datasets used for this work and the code (source and compiled) are available at http://cgs.csail.mit.edu/rulers. The array data is available at GEO under accession number GSE23524.

## Supporting Information

Figure S1 **Plot of predicted log-intensity vs distance for intervals of size 1000, 2000, 4000, and 8000.** The probability of termination at any base is.001 in all four intervals. Note the relatively linear shape over most of the interval followed by a more rapid decrease as the end effects become dominant.
(PNG)

Figure S2
**Ruler array data at a TY element insertion on chromosome XI in 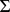
1278b.** Note the blue line, which indicates the channel ratio. To the right of the insertion, marked by the black arrow, the ratio is close to one. To the left of the insertion, the 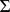
1278b probe intensities are lower than the S288c intensities and the ratio increases accordingly.(PNG)

Figure S3 **The four cases in which the Ruler Array analysis infers the presence of an indel from the segment fitting output.** In (a), the segment fitting used one segment to fit the green channel but two segments to fit the red channel; consequently, the analysis makes a call at the split point in the red channel. In (b), the segment fitting used two segments in each channel. The green channel is greater to the right of the break but of lower magnitude to the left. If the change is large enough, the analysis calls this boundary an indel. This change is commonly observed at AT repeat length changes. Example (c) illustrates another change common at repeat length or repetitive element changes. There is a segment boundary in both channels, but the intensities drop much more in one channel than the other. A restriction site, or the insertion of an element that contains a restriction site such as a TY, generates the signature seen in (d).
(PNG)

Table S1
**The 36 PCR-confirmed indels between S288C and 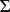
1278b used for Ruler Array validation.**
(TXT)

Text S1 **Supporting text one presents the the normalization methods and linefitting technique.**
(PDF)
