# Supplementary figures and images for: Ruler Arrays Reveal Haploid Genomic Structural Variation
Source: PLoS One. 2012 Aug 27;7(8):e43210. doi: 10.1371/journal.pone.0043210 (PMC3428316; doi:10.1371/journal.pone.0043210)

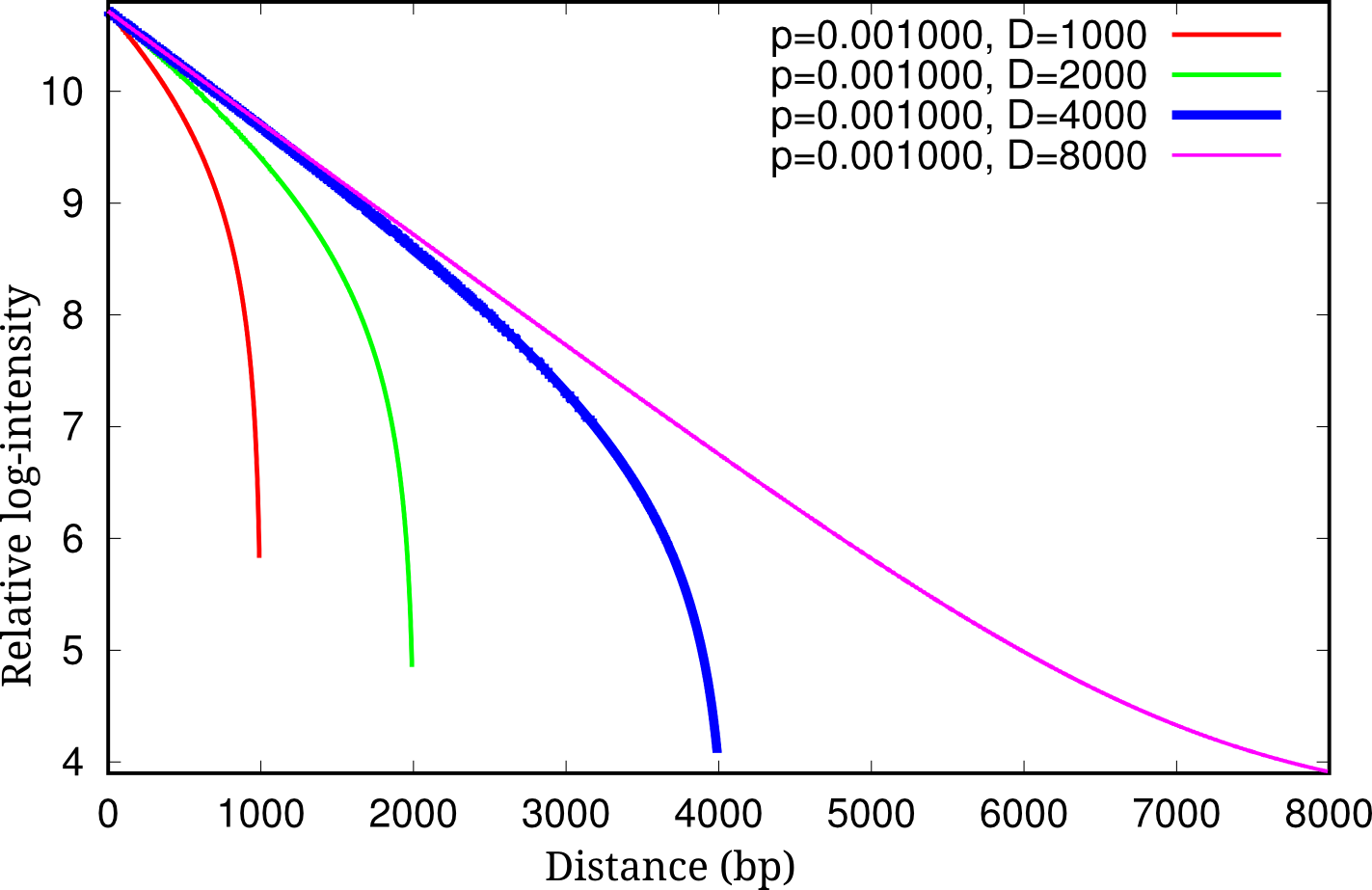

Supplement: Figure S1 — Plot of predicted log-intensity vs distance for intervals of size 1000, 2000, 4000, and 8000. The probability of termination at any base is.001 in all four intervals. Note the relatively linear shape over most of the interval followed by a more rapid decrease as the end effects become dominant. (PNG) [file pone.0043210.s001.png]

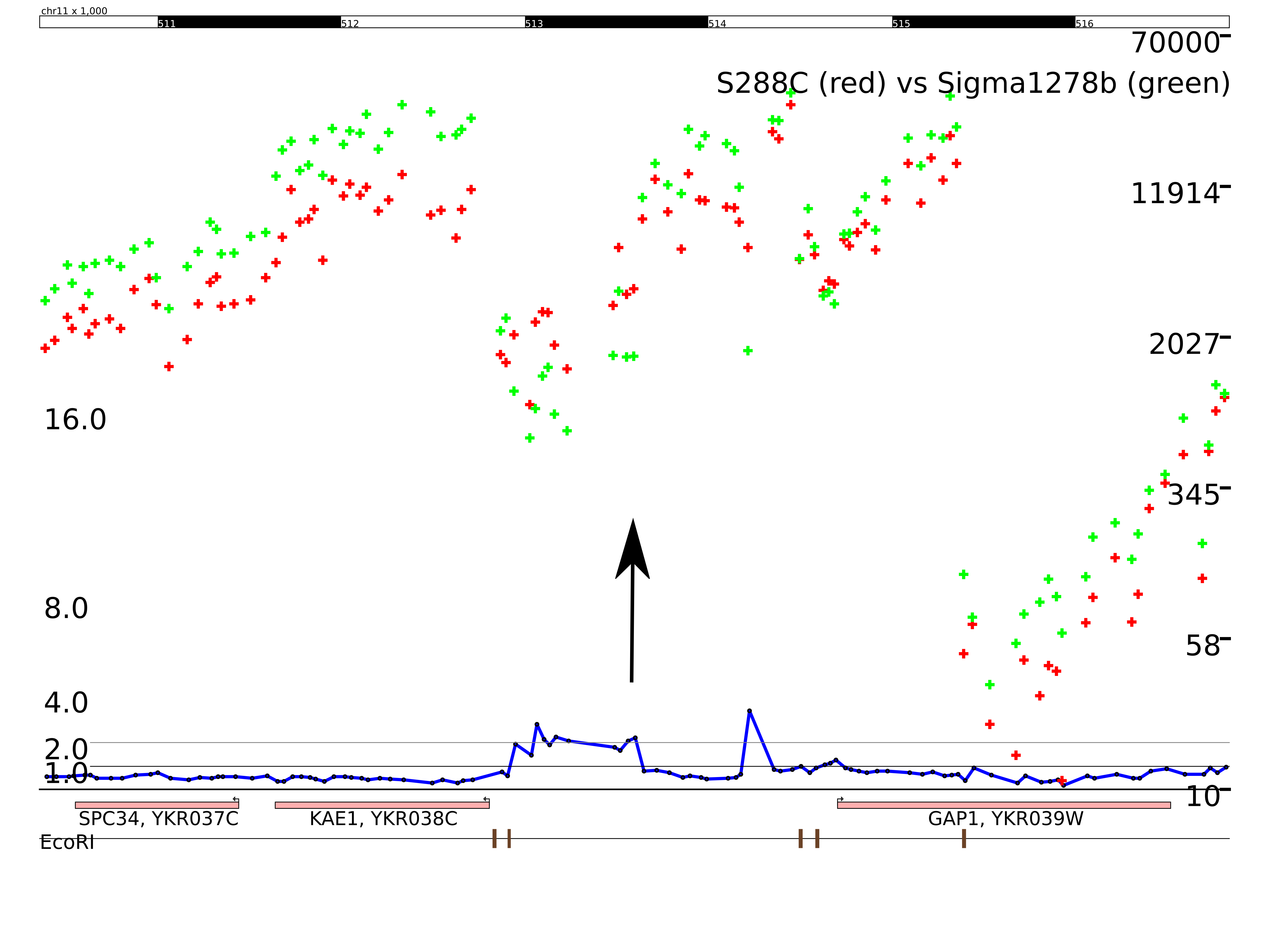

Supplement: Figure S2 — Ruler array data at a TY element insertion on chromosome XI in 1278b. Note the blue line, which indicates the channel ratio. To the right of the insertion, marked by the black arrow, the ratio is close to one. To the left of the insertion, the 1278b probe intensities are lower than the S288c intensities and the ratio increases accordingly. (PNG) [file pone.0043210.s002.png]

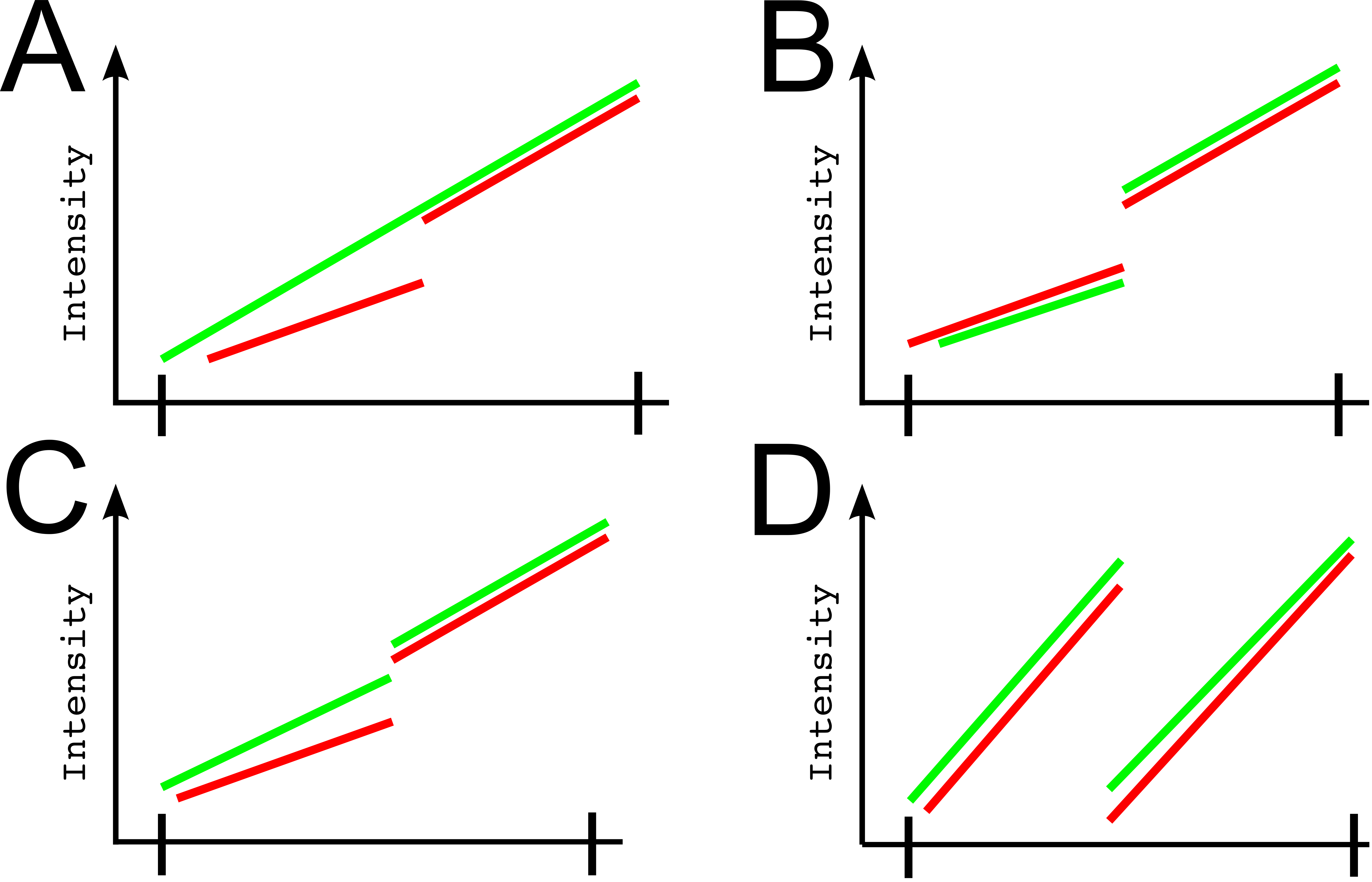

Supplement: Figure S3 — The four cases in which the Ruler Array analysis infers the presence of an indel from the segment fitting output. In (a), the segment fitting used one segment to fit the green channel but two segments to fit the red channel; consequently, the analysis makes a call at the split point in the red channel. In (b), the segment fitting used two segments in each channel. The green channel is greater to the right of the break but of lower magnitude to the left. If the change is large enough, the analysis calls this boundary an indel. This change is commonly observed at AT repeat length changes. Example (c) illustrates another change common at repeat length or repetitive element changes. There is a segment boundary in both channels, but the intensities drop much more in one channel than the other. A restriction site, or the insertion of an element that contains a restriction site such as a TY, generates the signature seen in (d). (PNG) [file pone.0043210.s003.png]
