# Supplementary material for: Ruler Arrays Reveal Haploid Genomic Structural Variation
Source: PLoS One. 2012 Aug 27;7(8):e43210. doi: 10.1371/journal.pone.0043210 (PMC3428316; doi:10.1371/journal.pone.0043210)
Supplement: Text S1 — Supporting text one presents the the normalization methods and linefitting technique. (PDF) [file pone.0043210.s005.pdf]

Supplementary Methods

Adapter Design

The adapter (and therefore primer) sequences were chosen to match the overhang left by EcoRI, have minimal genomic matches in yeast, and have a melting temperature suitable for the polymerase extensions. The sequence used was

```
P- AATTGGAGGAGGGAAGGGGG-B
        CCTCCTCCCTTCCCCC
```

Where P indicates a 5' phosphate (necessary for the ligation) and B indicates a 3' biotin to allow purification of the ligated material from the remainder of the reaction mix.

Note that the shorter oligo serves both as part of the adapter (in the ligation reaction) and as the primer in the polymerase extensions.

Data Preparation

Array Scanning and Feature Extraction Arrays were scanned on an Agilent microarray scanner (G2505B) and feature extracted with Agilent's software (9.5.3.1) to produce an AFE file.

Normalization We normalize the microarray data as follows. First, we compute the median intensity in each channel and then compute a correction factor as $f = \frac{\text{median}(Cy5)}{\text{median}(Cy3)}$. Then, the Cy5 values are adjusted as $Cy5' = Cy5 \cdot f$. This median normalization yields a dataset in which the median intensity is the same in both channels.

For one replicate, we used a crosstalk normalization designed to account for bleed of the Cy5 labeled nucleotides in the Cy3 channel and vice versa. For this procedure, we had previously computed the coefficients of crosstalk and adjusted the median normalized data for each probe as follows:

$$\begin{aligned} Cy5' &= (Cy5 - k_{Cy5,Cy3} \cdot Cy3)(1 - k_{Cy5,Cy3} \cdot k_{Cy3,Cy5}) \\ Cy3' &= (Cy3 - k_{Cy3,Cy5} \cdot Cy5)(1 - k_{Cy5,Cy3} \cdot k_{Cy3,Cy5}) \end{aligned}$$

Finally, we normalized all data with a linefitting normalization. This procedure assumes that the intensities in both channels should fall roughly along the line $Cy5 = Cy3$. As such, it performs linear regression on the observed data and then rotates the data such that the fitted line has slope one and intercept zero. This procedure is implemented in our SQL database with the following Java code and SQL statements.

```
stmt.execute("update " + dataTable + "
  set channelone = log(2,channelone), channeltwo = log(2,channeltwo)");

String sql = "select to_number(atan(REGR_SLOPE(channelone, channeltwo))"
+ "- atan(1)), to_number(REGR_INTERCEPT(channelone,channeltwo)) from "
+ dataTable + " where channelone - channeltwo < 3 and channelone -
channeltwo > -3";
```

```
ResultSet rs = stmt.executeQuery(sql);
rs.next();
factor = rs.getDouble(1);
intercept = rs.getDouble(2);

stmt.execute("update " + dataTable + " set channelone = channelone - " +
intercept);
stmt.execute("update " + dataTable + " set channelone = " +
"power(2,sqrt(channelone*channelone + channeltwo*channeltwo) * " +
"sin(atan(channelone/channeltwo) - " + factor + ")), " +
"channeltwo = power(2,sqrt(channelone*channelone + channeltwo*channeltwo) *
cos(atan(channelone/channeltwo) - " + factor + " ))");
stmt.execute("update " + dataTable + " set ratio = channelone /
channeltwo");
```

Computational Analysis

The ruler array computational method simultaneously performs linefitting (with weighted linear regression) and segmentation (to determine the boundaries of each fitted line) on both data channels. The procedure tries to use the same segments and the same parameters for both channels; it infers the presence of indels from segment boundaries present in one channel but not the other and from patterns of the parameters of the fitted lines.

Model

The linefitting and segmentation procedure minimizes the log-likelihood of the data and the parameters. The terms are

- the log-likelihood of the data.
  $$\Sigma \log(\frac{x_i - \hat{x}_i}{\sigma_i})$$
  where $\hat{x}_i$ is computed from the fitted line.
- whether the segments in the two channels have the same boundary. The prior probability of the segments using the same boundary helps determine the sensitivity of the analysis.
- whether the segments fitted to the two channels have the same slope. The prior probability of the segments having the same slope helps determine the sensitivity of the analysis.

Recursive Solution: One Channel

Let $f(a, b)$ be the result of fitting the function to the datapoints a through b where $1 \leq a \leq b \leq n$. $\mathcal{L}(f(a, b))$ is the log-likelihood of the observations given the values predicted by

the fit. Let $opt(a, b)$ be the optimal fit to the points $a..b$; the optimal fit may be either a single set of parameters for f or it may be a set of split points and the parameters for each interval between split points. The final goal is to find $opt(1, n)$. To find $opt(a, b)$, we use the following recursive procedure:

- First find $f(a, b)$ and its likelihood $\mathcal{L}(f(a, b))$. This is the "fit" outcome.
- For each $k : a \leq k < b$, recursively find the combined likelihood of $opt(a, k)$ and $opt(k + 1, b)$, $\mathcal{L}(opt(a, k)) + \mathcal{L}(opt(k + 1, b))$. Remember the k which gives the highest combined likelihood. This is the "split" outcome.
- Compare the result of fitting a single segment to $a..b$ to the result of fitting two segments with the boundary at the best k and choose whichever has the higher log likelihood. This gives $opt(a, b)$. If fitting gives the best result, then $\mathcal{L}(opt(a, b)) = \mathcal{L}(f(a, b))$. If the interval is split, then $\mathcal{L}(opt(a, b)) = \mathcal{L}(opt(a, k)) + \mathcal{L}(opt(k + 1, b))$.

Solution with Dynamic Programming

The recursive solution to $opt(1..n)$ is inefficient as it recomputes subproblems many times. For example, $opt(1..n)$ computes $opt(2..n)$, $opt(3..n)$, $opt(4..n)$, etc. $opt(2..n)$ computes $opt(3..n)$, $opt(4..n)$, etc. A more efficient approach computes $opt(i, j)$ only once and stores the result for future use.

The dynamic programming procedure computes $opt(a, b)$ for all a, b starting with the the smallest range of observations $a = b$ and works up to larger intervals. Since the two intervals $a..k$ and $k + 1..b$ are smaller than $a..b$ for all k, the solutions and log-likelihoods for those intervals will be ready when the procedure considers $a..b$.

Each solution $opt(a, b)$ is stored in a 2D table as it is computed, taking $O(n^2)$ space. Each element in the table stores either the parameters of the fit or the k at which the interval was split; each element (a, b) also stores $\mathcal{L}(opt(a, b))$. Filling the table will take at least $O(n^3)$ time since each of the $O(n^2)$ entries requires iterating over k; the exact runtime will depend on the time required to compute $f(a, b)$.

Segment Fitting with Linear Regression

As the log-intensities in a Ruler Array experiment fall roughly linearly as distance from a restriction site increases, we can use a simple linear model as f. For each interval a, b, the fitting procedure performs linear regression on the values from a to b, trying to predict the log intensity observed at position k as

$$\alpha + \beta \cdot \text{distance}(k, b)$$

For probes on the plus strand, the slope of the log intensities is positive and the distances are computed from the genomic position of probe b. For probes on the minus strand, the slope of the log intensities is negative and the distances are computed from a. All other aspects of the computation are the same for two strands.

We can estimate the standard deviation for an observation based on repeated observations of the same probe, observations of nearby probes, or a prior belief about the reliability of an observation given its intensity. Since our linear model fits the log-intensities, we perform the linear regression on the log-intensities and then exponentiate the predicted values before computing the log-likelihood:

$$\hat{x_i} = e^{\alpha+\beta\cdot\text{distance}(k,b)}$$

Under the assumption that the intensity observations for a probe are normally distributed around their mean, we can compute the probability for the probe intensity that the regression predicts given the observed intensity:

$$\begin{aligned}\mathcal{L}(f(a,b)) &= \Sigma_{k=a..b} log(p(\hat{x}_k|x_k,\sigma_k)) \\ &= \Sigma_{k=a..b} log(\frac{1}{\sigma\sqrt{2\pi}} e^{-\frac{(\hat{x_k}-x_k)^2}{2\sigma_k^2}})\end{aligned}$$

For the linear regression to maximize the log-likelihood, we use weighted linear regression. To maximize the log-likelihood, we must minimize

$$\Sigma_{k=a..b} \frac{(\hat{x_k}-x_k)^2}{2\sigma_k^2}$$

Linear regression finds the parameters to minimize

$$\Sigma_{k=a..b} (\hat{x_k}-x_k)^2$$

(the sum of the squares of the residuals). Weighted linear regression minimizes

$$\Sigma_{k=a..b} \frac{1}{w_k} (\hat{x_k}-x_k)^2$$

Thus setting the weights $w_k = \frac{1}{\sigma_k^2}$ makes the problems equivalent.

If X is the matrix of input variables with one column per variable and one row per datapoint. In the Ruler Array analysis, the first column is the constant one and the second column is the distance from the probe to the end of the interval. W are the weights, an $n \times n$ matrix in which $W_{kk} = w_k$ and all other entries are zero. Y is the vector of output observations. The parameters b are

$$b = (X^\top W X)^{-1} X^\top W Y$$

Runtime

The segment fitting algorithm performs two operations for every interval $a..b$ for $1 \leq a \leq b \leq n$:

1. Weighted linear regression to find the best single model for the entire interval.
2. A search over k to find the best point at which to split the interval.

Performing weighted linear regression on n observations with v variables requires time $O(v^2 n)$. The runtime breaks down as

- WY takes n multiplications
- $X^\top WY$ takes vn multiplications and at most $v(n-1)$ additions
- WX takes vn multiplications
- $X^\top WX$ takes $v^2 n$ multiplications and at most $v^2(n-1)$ additions.
- $(X^\top WX)^{-1}$ takes $O(v^2)$ operations

yielding an overall runtime of $O(v^2 n)$.

Since finding the combined log-likelihood in a split interval is computationally easy (the combined log-likelihood is the sum of the split log-likelihoods plus some prior), the linear regression dominates the work for each interval. The total runtime will thus be

$$\Sigma_{l=1}^{n}(n-l)O(lv^2) = O(v^2 n^3)$$

since there are $n-l$ intervals of length l. In our model, $v=2$, the constant and the distance from the probe to the end of the interval.

Jointly Handling Two Channel Experiments

We can extend the procedure to simultaneously handle two sets of observations (two channels) by adding more cases from which the choice with the highest log-likelihood is chosen. In particular, we add the option to fit both channels with the same parameters, fit both channels with different parameters, fit one channel but split the other, to split both at the same point, or to split both channels at different points. We use the subscripts 1 and 2 to indicate a fit or solution to data only from that channel.

The new log-likelihood choices are

Outcome	Log-Likelihood of Data	Log-Likelihood of Parameters
fit both<br>same parameters	$\mathcal{L}(f(a,b))$	$2 \cdot log(p_{\text{fit}}) + log(p_{\text{same params}})$
fit both<br>diff. parameters	$\mathcal{L}(f_1(a,b)) + \mathcal{L}(f_2(a,b))$	$2 \cdot log(p_{\text{fit}}) + log(1 - p_{\text{same params}})$
fit one<br>split one	$\mathcal{L}(f_1(a,b)) + \mathcal{L}(opt_2(a,k)) +$<br>$\mathcal{L}(opt_2(k+1,b))$	$log(P_{\text{fit}}) + log(1 - P_{\text{fit}}) +$<br>$log(1 - p_{\text{same params}})$
split both	$\mathcal{L}(opt(a,k)) + \mathcal{L}(opt(k+1,b))$	$2 \cdot log(1 - p_{\text{fit}})$<br>$log(1 - p_{\text{same params}})$

Variants on the fitting procedure might offer more possibilities, such as fitting the intervals with lines of the same slope but different intercepts. In practice, we found this variant

important as many ruler experiments include intervals in which the log-intensities in the two channels are the same shape (i.e. the same slope) but have different intercepts.

Calling Insertions and Deletions from Segment Fitting Output

As mentioned previously, a simple method for detecting insertions and deletions from the segment fitting output looks for the segment boundaries that exist only in one channel. In practice, we have augmented this procedure to recognize other patterns associated with indels.

Our best results have used joint segment fitting on both experimental channels. The "fit both channels with same parameters" outcome actually only fits both channels with the same slope, allowing the intercept to differ. This accounts for the observed phenomenon in which the absolute intensities differ but the shapes are similar; when analyzed in log-space, intensities that differ by some factor will be offset by some constant amount.

In addition to identifying single-channel segment boundaries as indels, the detection procedure identifies transitions from "fit both with same slope" to "fit with different slopes." This identifies regions in which the two channels are no longer the same or similar shapes. Many such points will be restriction sites, as changes in the character of the data often occur on restriction interval boundaries. The remainder ought to be insertions, deletions, or other events that change the data's character.

Finally, the analysis flags boundaries between segments at which the channel ratio changes dramatically or across which the intensity changes as it does at restriction sites.

Figure 3 shows the four cases in which the analysis calls an indel from the segment fitting.
